# Precise spatial structure impacts antimicrobial susceptibility of *S. aureus* in polymicrobial wound infections

**DOI:** 10.1073/pnas.2212340119

**Published:** 2022-12-15

**Authors:** Carolyn B. Ibberson, Juan P. Barraza, Avery L. Holmes, Pengbo Cao, Marvin Whiteley

**Affiliations:** ^a^School of Biological Sciences and Center for Microbial Dynamics and Infection, Georgia Institute of Technology, Emory-Children’s Cystic Fibrosis Center, Atlanta, GA 30310; ^b^Department of Microbiology and Plant Biology, University of Oklahoma, Norman, OK 73019

**Keywords:** *Pseudomonas aeruginosa*, *Staphylococcus aureus*, biogeography, chronic wounds, antibiotic resistance

## Abstract

A hallmark of microbial ecology is that interactions between members of a community shape community function. This includes microbial communities in human infections, such as chronic wounds, where interactions can result in more severe diseases. *Staphylococcus aureus* is the most common organism isolated from human chronic wound infections and has been shown to have both cooperative and competitive interactions with *Pseudomonas aeruginosa*. Still, despite considerable study, most interactions between these microbes have been characterized using in vitro well-mixed systems, which do not recapitulate the infection environment. Here, we characterized interactions between *S. aureus* and *P. aeruginosa* in chronic murine wounds, focusing on the role that both macro- and micro-scale spatial structures play in disease. We discovered that *S. aureus* and *P. aeruginosa* coexist at high cell densities in murine wounds. High-resolution imaging revealed that these microbes establish a patchy distribution, only occupying 5 to 25% of the wound volume. Using a quantitative framework, we identified a precise spatial structure at both the macro (mm)- and micro (µm)-scales, which was largely mediated by *P. aeruginosa* production of the antimicrobial 2-heptyl-4-hydroxyquinoline N-oxide, while the antimicrobial pyocyanin had no impact. Finally, we discovered that this precise spatial structure enhances *S. aureus* tolerance to aminoglycoside antibiotics but not vancomycin. Our results provide mechanistic insights into the biogeography of *S. aureus* and *P. aeruginosa* coinfected wounds and implicate spatial structure as a key determinant of antimicrobial tolerance in wound infections.

## Significance

Understanding of microbial interactions during infection often lacks biogeographical context, limiting understanding of community function. Using a mouse chronic wound model, we characterized the spatial structure of *P. aeruginosa* and *S. aureus* mono- and co-infections at the macro- and micro-scales. We discovered these bacteria coexist at high densities in chronic wounds, exhibiting a patchy distribution. Further, we quantified a precise spatial structure and found unlike bacterial burdens, spatial structure was dictated by location within the wound and dependent on a *P. aeruginosa*-secreted antimicrobial. Importantly, disruptions to the spatial structure altered *S. aureus* antibiotic tolerance. This work highlights the importance of microbial interactions for establishing the spatial structure in polymicrobial infections and implicates biogeography as a key determinant of antimicrobial efficacy.

Polymicrobial human infections are a major burden on human health. These infections are often more tolerant to antibiotics and have worse clinical outcomes compared to their single-microbe counterparts (1–7). Properties specific to polymicrobial infections are often attributed to interactions occurring between microbes, and much work has been done to identify and mechanistically understand these interactions (8–12). Recent evidence using preclinical infection models has shown that interactions between microbes impact the micron-scale spatial structure of the infecting community (13–16), implicating the spatial structure as a key component controlling community function, and thus infection outcomes (17). However, most of our understanding of polymicrobial interactions is derived from studies using in vitro models (13, 14). Hence, key elements of infection dynamics and the role of host factors are often overlooked.

*Pseudomonas aeruginosa* and *Staphylococcus aureus* are commonly used to study microbe–microbe interactions, both in vitro and in vivo (11, 13, 18–24). These microbes cooccur in several polymicrobial human infections, including chronic wounds and in the lungs of people with cystic fibrosis (1–3, 5, 25–29). There is conflicting evidence regarding the impact of coinfection on human disease outcomes, with some studies concluding that *P. aeruginosa* alone has worse outcomes (32–34) while others conclude that *P. aeruginosa–S. aureus* coinfections lead to more severe diseases (35–37). The experimental data are clearer in murine models of infection, which have shown that coinfection can result in increased antibiotic tolerance and worse infection outcomes (8, 10, 20, 28). While the mechanisms controlling these synergistic interactions are largely unknown in vivo, it has been hypothesized that *P. aeruginosa* and *S. aureus* occupy distinct regions in human chronic wounds (28), suggesting that biogeography may play a role in mediating polymicrobial wound infection outcomes.

Here, we collected more than 100 high-resolution confocal images of mouse chronic wounds infected with *P. aeruginosa* and *S. aureus* in mono- and co-infection. Using these images, we quantified the 3-dimensional macro- and micron-scale spatial structure of *P. aeruginosa* and *S. aureus* communities *in vivo* and defined the role of known *P. aeruginosa* extracellular antimicrobials on the spatial structure. We discovered that *S. aureus* and *P. aeruginosa* coexist in mouse wound infections at high bacterial densities, but their distribution is patchy. In addition, we discovered and quantified a precise, micron-scale spatial structure dependent on the *P. aeruginosa*-secreted small-molecule 2-heptyl-4-hydroxyquinoline N-oxide (HQNO) and that this spatial structure is different at the healing edge versus the center of the wound. Finally, we show that the community spatial structure has clinically important outcomes, including altered antibiotic tolerance.

## Results

### *S. aureus* and *P. aeruginosa* Coexist in a Mouse Surgical Wound Infection Model.

*S. aureus* and *P. aeruginosa* are commonly coisolated from human wounds (3, 29, 38). However, little is known about how the spatial structure of these communities and the interactions between these microbes impact clinical infection outcomes. To address this gap in knowledge, we used a murine surgical wound preclinical infection model (28). This model involves surgically removing a full-thickness area of skin from the shaved backs of mice (1.5 cm in diameter), applying a semipermeable bandage over the wound, and administering the bacterial inoculum to the wound topically underneath the bandage. We chose this model for several reasons: It is a self-resolving infection model that can be monitored for up to 3 wk; the wound material can be easily excised and immediately studied using microbiological assays and confocal imaging; and recent work from our lab indicates that this model accurately recapitulates *P. aeruginosa* gene expression signatures observed in human chronic infections (39).

**Fig. 1.**
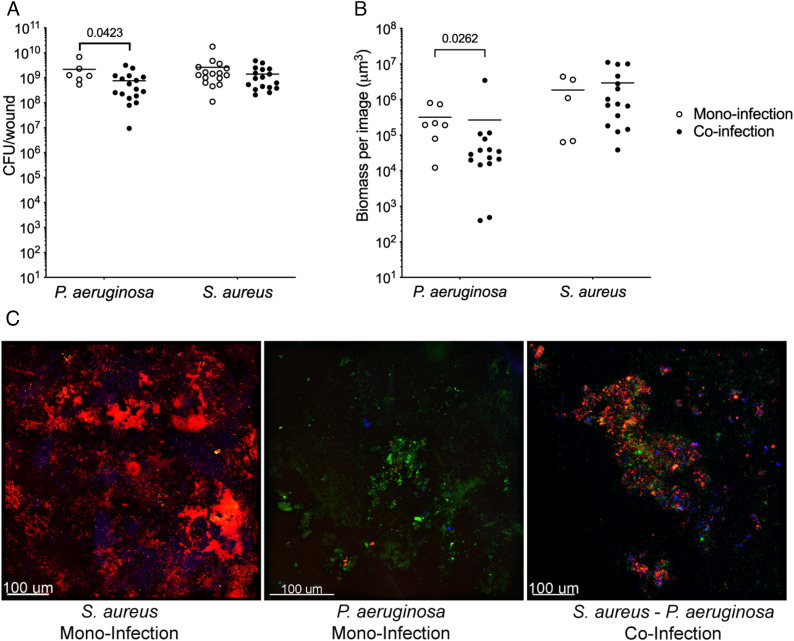
*S. aureus* and *P. aeruginosa* coexist in a murine surgical wound model. (*A*) Bacterial burdens in mono- (open circles) and co-infected (closed circles) murine chronic wounds 4 d post infection. Number of animals used was as follows: *P. aeruginosa* monoinfection n = 6, *S. aureus* monoinfection n = 16, *S. aureus*/*P. aeruginosa* coinfection n = 17. (*B*) Bacterial biomass measured by confocal microscopy of murine wounds infected with *S. aureus* or *P. aeruginosa* alone (monoinfection, open circles) or together (coinfection, closed circles). *P*-values shown were determined using a Mann–Whitney test. (*C*) Confocal microscopy of murine wounds infected with *S. aureus* (red, first panel) or *P. aeruginosa* in monoinfection (green, second panel) or in coinfection (third panel) at 4 d postinfection. Host cells (blue) were stained with NucBlue in the mounting medium.

Coinfection of murine surgical wounds with the community-associated methicillin-resistant USA300 *S. aureus* strain LAC and *P. aeruginosa* strain PA14 revealed that these bacteria coexist at equivalent and high bacterial burdens (~10^9^ colony-forming units (CFU)/wound) after 4 d (Fig. 1*A*). *S. aureus* coinfection numbers were equivalent to those observed in monoinfection (Fig. 1*A*), indicating that coinfection does not impact *S. aureus* fitness in the wound. However, *P. aeruginosa* numbers during coinfection were significantly lower than those in monoinfection, although the differences were quantitatively small (~2.5-fold). These findings were confirmed by assessing total biomass with confocal microscopy in wounds infected with *S. aureus* and *P. aeruginosa* that constitutively express dsRed or GFP, respectively (Fig. 1 *B* and *C* and *SI Appendix*, Fig. S1). We also found that coexistence is not specific to *P. aeruginosa* PA14, as *S. aureus* LAC and the *P. aeruginosa* human chronic wound clinical isolate CW2-B1 similarly coexist in wounds (*SI Appendix*, Fig. S2).

### Macroscale Differences in the Spatial Distribution of *P. aeruginosa* in Coinfected Murine Chronic Wounds.

**Fig. 2.**
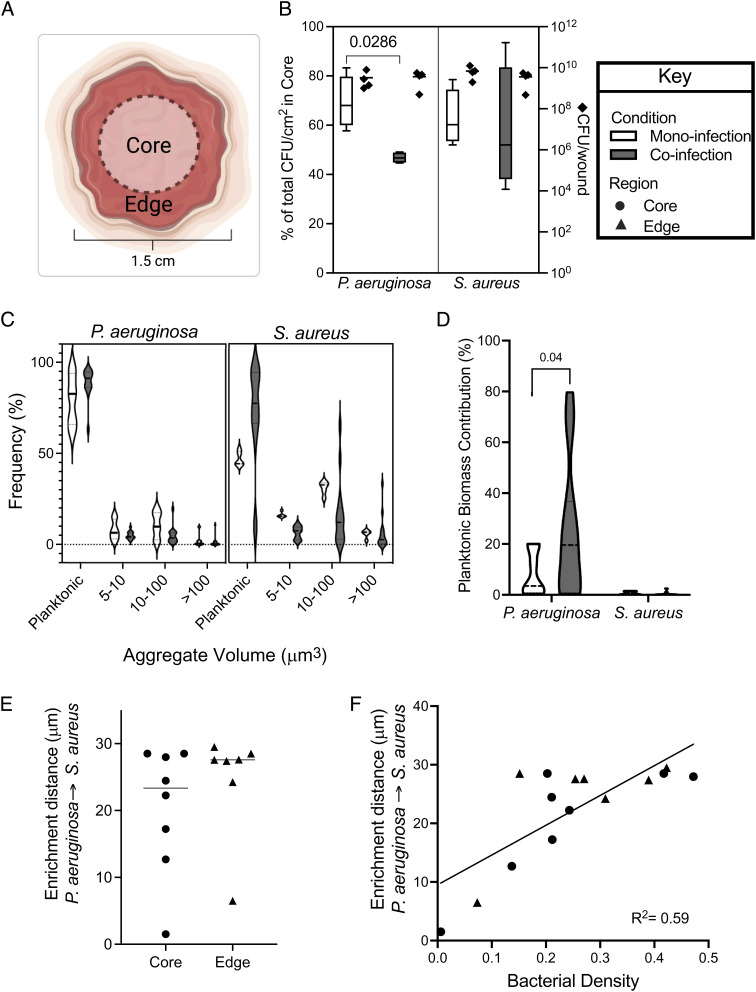
Characterization of *S. aureus* and *P. aeruginosa* biogeography and spatial distribution in murine wounds. (*A*) Diagram of the core and edge regions of a mouse wound. (*B*) Percent of the total *P. aeruginosa* and *S. aureus* CFU normalized by surface area found in the core of mono- (open bar) or co-infected (closed bars) wounds (left ordinate). Total bacterial CFU of each wound (diamond) is plotted on the right ordinate. Statistical significance was determined with a Mann–Whitney test. Four animals were used per condition. (*C*) Frequency of *P. aeruginosa* or *S. aureus* objects in each size bin in the core of mono- (open) and co-infected (closed) murine chronic wounds. (*D*) Percentage that planktonic cells contribute to the total bacterial biomass in mono- (open) and co-infected (closed) wounds. Statistical significance was determined using a Student’s *t* test. (*E*) Enrichment distance, defined as the distance in which proportional occupancy of *S. aureus* from *P. aeruginosa* is highest, in the core (circle) or edge (triangle) of murine wounds coinfected with *S. aureus* and *P. aeruginosa*. (*F*) Relationship between bacterial density and enrichment distance in the core (circle) or edge (triangle) of murine wounds coinfected with *S. aureus* and *P. aeruginosa*. R^2^ was determined by a nonlinear best-fit line.

Recent work indicates that in addition to bacterial burden, an important contributor to infection severity and outcome is the spatial arrangement of bacteria (15, 16). While this previous work focused on the micron-scale spatial structure in mouse abscess infections and human dental caries, there is anecdotal evidence that microbes in human wounds also spatially organize (28). To test this, we first assessed the spatial organization of *S. aureus* and *P. aeruginosa* in mono- or co-infected wounds at the microbial macroscale (mm-level resolution) by dividing the wound into two regions: the center core and the outer edges (Fig. 2*A*). This was accomplished by taking a 10-mm punch biopsy from the center of each wound and quantifying bacterial numbers of both the center core (inside the punch) and the healing edge (outside the punch). While there was no difference in the distribution of *S. aureus* between mono- and co-infection, *P. aeruginosa* was found at lower levels in the core during coinfection (Fig. 2*B*, Mann–Whitney test, *P* = 0.028). In addition, there was less variance in the percentage of *P. aeruginosa* in the core during coculture compared to monoculture infections in the wounds. These data indicate that the presence of *S. aureus* causes a relocalization of *P. aeruginosa* to the outer edges of the wound, and this spatial structure is highly conserved.

### Micron-Scale Differences in Spatial Distribution in Coinfected Murine Chronic Wounds.

We next assessed the micron-scale spatial structure of mono- and co-infected wounds using high-resolution confocal microscopy. Wounds were initially broadly surveyed for bacterial fluorescence, revealing that most of the wound volume is not occupied by bacteria. Instead, both bacteria showed a heterogeneous distribution of biomass, represented as patches of varying size and abundance (*SI Appendix*, Fig. S3). We next leveraged the confocal images to determine whether bacteria existed as planktonic (individual) cells or aggregates (biofilms). We focused on planktonic and aggregated bacteria as it is well known that they have distinct phenotypes that likely alter host–microbe interactions and infection outcomes (40–41). Confocal images of the center core and edges of mono- and co-infected wounds were analyzed as previously described (13) to quantify the number of *P. aeruginosa* and *S. aureus* present planktonically (cell volume < 5 μm^3^) and in aggregates (Fig. 2*C*). In addition, cells present in aggregates were classified by size using the following ranges: 5 to 10 μm^3^, 10 to 100 μm^3^, >100 μm^3^.

Fig. 2*C* shows that on average 71% of the *S. aureus* objects (comprising both planktonic cells and aggregates) observed in coinfection are planktonic cells, which is higher than that observed in monoculture (47%, Mann–Whitney test *P* = 0.014). This increase in relative frequency of planktonic cells in coinfection occurred along with a decrease in aggregates of the first two size ranges. Consequently, the relative frequency of aggregates in the 5 to 10 μm^3^ and 10 to 100 μm^3^ size ranges was higher, 16% and 31%, for *S. aureus* in monoinfection compared to coinfection, 3% and 4%, respectively (Mann–Whitney test, *P =* 0.0001 and 0.014, respectively). However, while there were more *S. aureus* planktonic cells in coinfected wounds, these cells still contributed only ~1% of the total biomass of *S. aureus* in the wounds (Fig. 2*D*), indicating that aggregates dominate both mono- and co-infected wounds. No differences in planktonic numbers or aggregate size were observed for *S. aureus* present in the wound center or on the edges.

The number of *P. aeruginosa* planktonic cells and aggregates was similar in the core and edges of the wound (Fig. 2*C*). As observed with *S. aureus*, there were more *P. aeruginosa* planktonic cells than aggregates in both mono- and co-infected wounds. However, *P. aeruginosa* planktonic cells comprised a larger proportion of the microbial biomass compared to *S. aureus*, constituting 8% of the biomass in monoinfection, which increased to 25% in coinfection (Fig. 2*D*, Student’s *t* test *P* = 0.04). These results reveal that while *P. aeruginosa* exists primarily as aggregates in both mono- and co-infected wounds, planktonic cells are present, and their number increases in the presence of *S. aureus*.

To further characterize the spatial structure of *P. aeruginosa* and *S. aureus* in wounds, we quantified the micron-scale spatial structure of the community using a computational approach recently developed by our lab (13). This approach begins by focusing on one species within a community and calculating the proportional occupancy (PO), which quantifies the composition of the immediate surroundings of a focal species in relation to other community members at various distance intervals in three dimensions at the micron scale. Here, *P. aeruginosa* was used as the focal species, and PO of *S. aureus* relative to 1000 randomly selected *P. aeruginosa* voxels was calculated for each image. This analysis revealed a positive correlation of distance with PO, suggesting an active segregation mechanism, similar to findings from our previous work using an in vitro cystic fibrosis preclinical model (13). As we saw evidence of an active segregation mechanism, we next calculated the *S. aureus* enrichment distance. Here, the enrichment distance is defined as the distance from *P. aeruginosa* (focal species) at which the PO of *S. aureus* is highest. Thus, enrichment distance indicates where *S. aureus* biomass is over-represented relative to *P. aeruginosa*. We found the median enrichment distance of *P. aeruginosa* to *S. aureus* was similar in the wound core (20.4 ± 9.5 µm SEM) and in the wound edge (24.5 ± 8.1 µm SEM) (Fig. 2*E*). We next determined if there was a relationship between local bacterial density and enrichment distance, testing the hypothesis that bacteria will be closer together in areas of higher bacterial density. Our data revealed a positive correlation between enrichment distance of *S. aureus* from *P. aeruginosa* and increased bacterial density (Fig. 2*F*, nonlinear best-fit line R^2^ = 0.59), indicating that community spatial structure changes as the density of the microbial community increases, with greater segregation of *P. aeruginosa* and *S. aureus* in highly dense communities.

### HQNO and Pyocyanin Do Not Impact Bacterial Fitness or Macroscale Spatial Distribution in Murine Wounds.

**Fig. 3.**
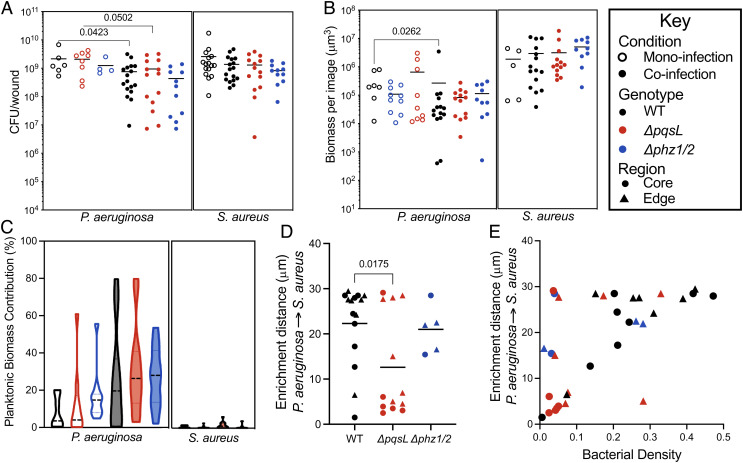
Role of HQNO and pyocyanin on bacterial fitness and spatial distribution in murine wounds. (*A*) CFU per wound of *P. aeruginosa* or *S. aureus* in mono- (open circles) and co-infection (closed circles) with wildtype (black), *ΔpqsL* (red), or *Δphz1/2* (blue). *P*-values were determined using a Mann–Whitney test. (*B*) Total biomass determined by confocal microscopy of wounds in mono- (open) or co-infection (closed) with *P. aeruginosa* WT (black), *ΔpqsL*(red), or *Δphz1/2* (blue) mutants. (*C*) Percentage that planktonic cells that contribute to the total bacterial biomass in wounds in mono- (open) or co-infection (closed) with *S. aureus* and *P. aeruginosa* WT (black), *ΔpqsL*(red), or *Δphz1/2* (blue) mutants. (*D*) The enrichment distance of *P. aeruginosa* from *S. aureus* in microns. Measurements from images taken from the wound core are shown with circles, while images taken from the wound edge are shown with triangles. *P*-value was determined by a Student’s *t* test. (*E*) Relationship between bacterial density and enrichment distance in the core (circles) or edge (triangles) of murine wounds coinfected with *S. aureus* and *P. aeruginosa* WT (black), *ΔpqsL*(red), or *Δphz1/2* (blue).

Many interactions between *P. aeruginosa* and *S. aureus* are thought to be hostile, and these interactions have been shown to be mediated through *P. aeruginosa*-secreted molecules. Two *P. aeruginosa*-secreted molecules with antimicrobial activity, pyocyanin and HQNO, have been of particular interest as they impact *S. aureus* physiology and fitness during in vitro coculture (13, 42–46). To test whether these antimicrobials impact *P. aeruginosa*–*S. aureus* fitness and spatial structure, we coinfected wounds with WT *S. aureus* and isogenic *P. aeruginosa* mutants that are unable to produce pyocyanin or HQNO. The pyocyanin mutant, *P. aeruginosa* Δ*phz1/2*, contains deletions of both *phzA-E* operons and has been previously characterized (47). The HQNO mutant, *P. aeruginosa* Δ*pqsL,* contains a deletion of *pqsL* and is thus defective in the final step in HQNO biosynthesis. This mutant was also previously characterized (13), and transcriptome analysis of Δ*pqsL* and WT *P. aeruginosa* in the wound model revealed that deletion of *pqsL* does not have polar effects on surrounding genes (*SI Appendix*, Table S2). We discovered that there was no difference in bacterial numbers when mice were coinfected with *S. aureus* and either WT *P. aeruginosa* PA14 or the isogenic mutants (Fig. 3 *A* and *B*), and similar results were observed in *S. aureus* coinfections with the *P. aeruginosa* wound isolate CW2-B1 or its isogenic *pqsL* mutant (*SI Appendix*, Fig. S2). We also compared the contribution of planktonic cells to the overall biomass of *S. aureus* and *P. aeruginosa*, revealing no differences in coinfections containing WT *P. aeruginosa* PA14 or the isogenic mutants (Fig. 3*C*). These data indicate that HQNO and pyocyanin do not impact fitness or growth mode (planktonic/aggregate) of *S. aureus* or *P. aeruginosa* in coinfected wounds.

### HQNO But Not Pyocyanin Alters the Micron-Scale Spatial Structure of *S. aureus* and *P. aeruginosa* in Wounds.

We next tested the role that HQNO and pyocyanin play in the micron-scale spatial structure of coinfecting communities. To accomplish this, we calculated the enrichment distance of *S. aureus* from *P. aeruginosa* in chronic wounds coinfected with *S. aureus* and *P. aeruginosa ∆pqsL* or *∆phz1/*2 and compared this to coinfections with WT *P. aeruginosa* (Fig. 3*D* and *SI Appendix*, Fig. S1). Coinfection with *P. aeruginosa ∆pqsL* significantly reduced the *S. aureus* enrichment distance (12.6 ± 3.1 μm SEM) compared to coinfection with WT *P. aeruginosa* (22.2 ± 2.3 μm SEM, unpaired Student’s *t* test *P* = 0.03). No differences in enrichment distance were observed for coinfection with *P. aeruginosa ∆phz1/*2. These data indicate that the micron-scale spatial structure of the *P. aeruginosa–S. aureus* microbial community in a mouse wound is impacted by HQNO but not pyocyanin.

Is the impact of HQNO on the micron-scale spatial structure dependent on the local density of the microbial community? One possibility is that *S. aureus* is closer to *P. aeruginosa ∆pqsL* than WT *P. aeruginosa* during coinfection simply due to differences in localized density of the microbial patches in the infection site. To test this, we compared the enrichment distance of *S. aureus* from *P. aeruginosa* WT and *∆pqsL* as a function of local bacterial density. These data revealed that *P. aeruginosa ∆pqsL* coinfections were generally less dense than WT *P. aeruginosa* coinfections, and unlike WT *P. aeruginosa* coinfections, the enrichment distance was not highly correlated with density (Fig. 3*E*). These data indicate that *S. aureus* is not closer to *P. aeruginosa ∆pqsL* because of a general increase in localized bacterial density in the wounds.

### HQNO Enhances *S. aureus* Tolerance to Aminoglycosides during Wound Coinfection.

**Fig. 4.**
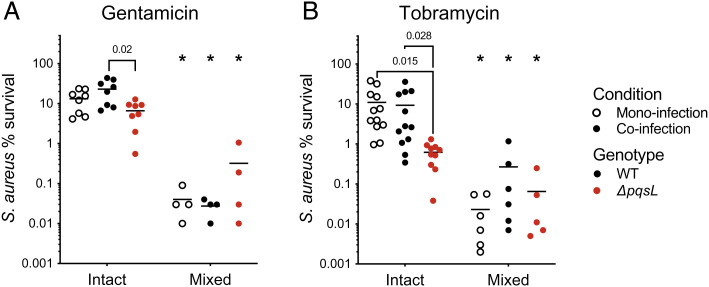
*S. aureus* aminoglycoside tolerance in intact and mixed wounds. Percent survival of *S. aureus* to gentamicin (*A*) or tobramycin (*B*) in intact (structured) or mixed (homogenized) wounds compared to nonantibiotic treated portions of the same wound. Monoinfection is shown with open circles, coinfection with WT *P. aeruginosa* PA14 with closed circles, and coinfection with *P. aeruginosa ΔpqsL* with red circles. All *P*-values were determined by a Mann–Whitney test; asterisks indicate *P* < 0.01 comparing intact and mixed wounds. The following number of animals were used: Panel *A*: *S. aureus* monoinfection, n = 4; *S. aureus*/*P. aeruginosa* PA14 WT coinfection, n = 4; *S. aureus*/*P. aeruginosa* PA14 *ΔpqsL* coinfection, n = 4. Panel *B*: *S. aureus* monoinfection, n = 6; *S. aureus*/*P. aeruginosa* PA14 WT coinfection, n = 6; *S. aureus*/*P. aeruginosa ΔpqsL* coinfection, n = 5.

Although it is clear that HQNO impacts the micron-scale spatial structure of coinfected wounds, an open question is whether this results in community functional changes. Previous studies have shown that *P. aeruginosa*-produced HQNO increases the tolerance of *S. aureus* to aminoglycosides in vitro (45); thus, we focused on this phenotype. We tested two different aminoglycosides: gentamicin, which has been used to successfully treat wound infections (49, 50), and tobramycin, which is commonly used to treat chronic lung infections and more recently has been pursued as a prophylactic treatment to prevent wound infections (51, 52). We first tested if *S. aureus* had altered tolerance in coinfected wounds and the role that HQNO plays in aminoglycoside tolerance. This was accomplished by exposing mono- and co-infected wounds to a high dose of either gentamicin (200 μg/ml, >100X *S. aureus* MIC) or tobramycin (512 ug/ml, >75X *S. aureus* MIC) for 1 h. We found that while there was no difference in *S. aureus* tolerance to either aminoglycoside when alone or in coinfection with WT *P. aeruginosa* PA14, there was a significant decrease in tolerance during coinfection with *P. aeruginosa ΔpqsL* (Fig. 4). In contrast, we saw no difference in *S. aureus* tolerance to vancomycin during coinfection with WT or *ΔpqsL* (*SI Appendix*, Fig. S4*A*)*.* This reduction in *S. aureus* aminoglycoside tolerance was also observed during coinfection with the *P. aeruginosa* strain CW2-B1 *ΔpqsL* strain compared to coinfection with WT *P. aeruginosa* CW2-B1 (*SI Appendix*, Fig. S4*B*). These data indicate that the production of HQNO is a key mediator of *S. aureus* antibiotic tolerance during coinfection.

To test if aminoglycoside tolerance was dependent on the spatial structure, wounds were quickly homogenized to eliminate the spatial structure using large steel beads that break up bacterial aggregates but do not impact viability. Mixed (homogenized) wound communities showed a 10 to 1000-fold increase in killing to both gentamicin and tobramycin compared to intact samples (Fig. 4). Finally, we confirmed our in vivo findings (*SI Appendix*, Fig. S5) using a chronic cystic fibrosis infection model that also supports precise spatial patterning of *P. aeruginosa–S. aureus* cocultures (13, 53). As in the murine wounds, *S. aureus* tolerance to tobramycin was reduced during coculture with *ΔpqsL* compared to WT *P. aeruginosa* and further reduced when cocultures were vigorously mixed (*SI Appendix*, Fig. S5). Importantly, the addition of exogenous HQNO increased tobramycin tolerance in the well-mixed cultures. These data indicated that the spatial structure enhances *S. aureus* tolerance to aminoglycosides and that this effect is eliminated by modifying the microbial spatial structure either mechanically (homogenization) or by altering the interactions between community members (absence of HQNO).

## Discussion

Polymicrobial interactions are proposed to be a fundamental process affecting human health and disease. There is recent evidence suggesting that the micron-scale spatial structure resulting from metabolic interactions between bacterial species is a key determinant of the function of polymicrobial infection communities, although the in vivo data are limited to less than a handful of preclinical studies (15, 16, 54, 55). Here, we discovered that two coinfecting microbes that don’t coexist under many laboratory conditions stably coinfect murine surgical wounds at high densities and establish a precise micron-scale spatial structure that impacts antibiotic susceptibility. The fact that this spatial structure is controlled by the *P. aeruginosa* antimicrobial HQNO furthers our understanding of the role of this small molecule in mediating polymicrobial interactions, extending in vitro results into a mouse model that has been quantitatively shown to recapitulate the gene expression of *P. aeruginosa* during human chronic infection (39). Further, that the production of pyocyanin has no observed impact on spatial structuring of this community reveals that not all known *P. aeruginosa*-produced antimicrobials are critical for the emergence of the precise spatial structure observed. While an alternative explanation is that pyocyanin is not produced in this infection model, we find this unlikely as genes involved in pyocyanin production show increased or equivalent expression in this murine model compared to an in vitro model in which pyocyanin is produced at detectable levels (56). Regardless, our data support a critical role for HQNO in modulating the *P. aeruginosa*–*S. aureus* community structure in a chronic infection environment.

An important aspect of this work was the acquisition of large numbers of high-resolution images of infected wounds. Approximately 1 to 3% of each wound was imaged at high resolution in our study, thus providing the opportunity to assess the community spatial structure at multiple scales. Bacterial growth within the wounds is patchy, as the size of groups of contiguous bacteria varies by more than three orders of magnitude (Fig. 2*C* and *SI Appendix*, Fig. S3) and localized areas of high density are spatially organized at the micron-scale (Fig. 2*F*). This is likely due to local host environmental conditions as wounds in this model are initially colonized by planktonic bacteria distributed over the entire surface of the wound. Thus, this patchy distribution likely emerges from these randomly distributed planktonic cells. We found it surprising that the enrichment distance between *P. aeruginosa* and *S. aureus* increased as the overall bacterial density increased, as we naively thought that higher densities would lead to closer association between these bacteria. Instead, our data support a model in which higher density within localized patches increases competition, which in turn leads to increased segregation.

Cells exist both planktonically and as aggregates in the wounds, although most of the bacterial biomass is present in aggregates. One of the more interesting discoveries here is that the presence of *S. aureus* drives more of the *P. aeruginosa* biomass into planktonic cells, with ~25% of the biomass present as planktonic cells during coinfection compared to 8% in monoinfection. The mechanism controlling this planktonic shift in *P. aeruginosa* is not known and could be a result of the direct interactions between *S. aureus* and *P. aeruginosa* or a change in the infection environment during coinfection. Irrespective of the mechanism, this is not a universal phenotype of *P. aeruginosa*–*S. aureus* coculture as it was not observed in an in vitro preclinical model of CF infection (13).

The observation that *S. aureus* grows in closer proximity to *P. aeruginosa ΔpqsL* than the WT (Figs. 2*E* and 3*D*) suggests that HQNO likely plays an antimicrobial role in the wound, causing *S. aureus* to colonize at a further distance from *P. aeruginosa*. Overall, the spatial structures of wound coinfections were different from those observed in the preclinical model of CF infection, in which these bacteria showed enrichment distances of less than 8 μm and *S. aureus* grew closer to the WT *P. aeruginosa* than to *ΔpqsL* (13). These results indicate that HQNO has differential effects on the spatial structure dependent on the environment and suggest that this molecule does not always serve an antimicrobial function.

Previous in vitro experiments showed HQNO enhances *S. aureus* tobramycin tolerance in vitro (45), and our data confirm this phenotype in coinfected murine chronic wounds using *P. aeruginosa* strain PA14 and the chronic wound clinical strain CW2-B1 (Fig. 4 and *SI Appendix*, Fig. S4*B*). The most interesting discovery from this study relates to the impact of spatial structure on gentamicin and tobramycin tolerance, supported by the observation that mechanical mixing of wound tissue results in identical *S. aureus* tolerance, whether in coculture with WT or *ΔpqsL P. aeruginosa* (Fig. 4). For these experiments, it was critical to use a mechanical mixing procedure that can be accomplished quickly and does not kill bacteria. While previous in vitro experiments implicate the *P. aeruginosa–S. aureus* spatial structure as a key determinant in antibiotic tolerance (57), to our knowledge, similar experiments have not been performed in vivo.

Confirmation of the in vivo wound findings with those using a preclinical model of chronic CF infection (*SI Appendix*, Fig. S5) further supports the conclusion that the HQNO-mediated spatial structure plays a role in aminoglycoside tolerance (Fig. 4 and *SI Appendix*, Fig. S5). Collectively, these data support a model in which *S. aureus* actively positions itself in a ‘sweet spot’ relative to *P. aeruginosa* that provides exposure to sublethal HQNO levels that enhance tolerance. Additionally, as mentioned above, this beneficial spatial positioning is clearly dependent on the environment as the wound and CF in vitro model have distinct spatial patterning (Fig. 3) (13). Finally, HQNO-mediated tolerance does not apply to all antimicrobials, as no impact was observed with vancomycin (*SI Appendix*, Fig. S4*A*).

We speculate that our results have relevance to human chronic wounds, as it has been reported that *P. aeruginosa* and *S. aureus* spatially segregate in these infections (28). Previous transcriptome data found that *pqsL* was expressed in 11/13 human-derived samples, indicating that HQNO is likely produced during human infection, including chronic wounds (39). While it is difficult to perform direct comparisons due to the lack of quantitative spatial structure data in human chronic wounds, collectively these studies suggest that *P. aeruginosa* and *S. aureus* spatially segregate in wound infections. Future studies will focus on using the quantitative spatial structure data generated in this study as a benchmark for comparison to in vitro models and human wounds using a similar framework to that recently developed in our laboratory using transcriptomic data (39, 58, 59).

## Materials and Methods

### Bacterial Strains and Growth Conditions.

Bacterial strains used in this study are listed in *SI Appendix*, Table S1. The *P. aeruginosa* clinical isolate, CW2-B1, was isolated from a chronic wound on the toe of a 78-y-old patient by the Clinical Microbiology Department at Nottingham University Hospitals (60). *S. aureus* strain LAC (AH1263) that constitutively expresses dsRed was created by moving pHC48 (61) from *S. aureus* strain RN4220 + pHC48 (AH3856) with staphylococcal bacteriophage ϕ11 via transduction. *P. aeruginosa* constructs that constitutively express GFP were generated by moving pBK-miniTn7-gfp2 into PA14, PA14 *∆pqsL,* and PA14 *∆phz1/2* via conjugation with the helper plasmids pUXBF13 and pRK2013 as previously described (48). *P. aeruginosa* strain CW2-B1 *∆pqsL* was constructed by allelic replacement using plasmid pEXG2pq via conjugation as previously described (13). All constructs were checked by PCR. Cultures were grown in brain–heart infusion broth at 37 °C under shaking at 225 rpm. and a flask-to-media volume ratio of ~7:1. Deletion of *pqsL* in strain *P. aeruginosa* PA14 *∆pqsL* was confirmed to be nonpolar as it did not impact expression of surrounding genes (*SI Appendix*, Table S2) and was previously genetically complemented (13).

### Chronic Surgical Wound Model.

Murine surgical wound infections were performed with 8- to 10-wk-old female C57BL/6 mice (Charles River), as previously described (9, 37, 56). Approximately 5 × 10^5^ CFU were used for each inoculum, with *S. aureus* and *P. aeruginosa* mixed at a ratio of 1:1 prior to inoculating for all coinfections (~2.5 × 10^5^ CFU of each species). Wound tissue was excised at 4 d postinfection. At least three biological replicates were used per condition in this model, which was determined from previous and preliminary data to be sufficient to yield statistically significant differences. For bacterial burdens, wound tissue was added to BeadBug tubes containing 1-mm steel beads and 850 µL of sterile phosphate buffered saline, for a total volume of ~1 ml. Tubes were bead beat one time for 30 s to homogenize tissue and were serially diluted with sterile PBS prior to enumeration by plate counts on mannitol-salt phenol red agar (MSA) and/or pseudomonas isolation agar (PIA). Plates were incubated at 37 °C overnight prior to determining the CFU on each plate.

### Quantifying Macroscale Spatial Structure.

For macrospatial structuring, a 10-mm biopsy punch was used to separate the center of the wounds from the outside after wound excision, leaving 2 pieces with surface area of approximately 0.79 cm^2^ and 0.98 cm^2^, for inside and outside, respectively. Bandages were then removed from each piece, and the resulting 4 pieces (inside tissue, inside bandage, outside tissue, and outside bandage) were weighed and added to BeadBug tubes containing 1-mm steel beads and PBS to a total volume of 1 ml. Samples were then bead beat for 30 s, serially diluted in PBS, and enumerated on MSA and PIA plates as described above.

### Antibiotic Tolerance.

Following excision, wounds were divided into 4 equal quarters with sterile surgical scissors. One piece from each wound was added to 3 ml of PBS alone, and two pieces of each wound were added to 3 ml of PBS containing 512 µg/ml tobramycin or 200 µg/ml gentamicin in a 12-well microtiter plate (Corning Costar). The remaining piece was added to a BeadBug tube containing PBS and 1-mm steel beads and bead beat for 30 s prior to the addition of tobramycin at a final concentration of 512 ug/ml or gentamicin (200 µg/ml). Plate and tubes were incubated for 1 h at 37 °C. Tissue pieces in the plate were transferred to a fresh microtiter plate containing only PBS prior to transferring the pieces to BeadBug tubes and bead beating for 30 s to homogenize. The tube containing homogenized tissue was spun for 4 min at max speed to pellet cells and tissue fragment; the supernatant was removed and replaced with 900 µL of fresh PBS and vortexed for 30 s to resuspend. All tubes were then serially diluted in PBS, and bacteria were enumerated by plating on MSA and PIA as described above. For the in vitro experiments, synthetic CF sputum media (SCFM2) cocultures were prepared as outlined previously (13), and 300 µL culture volumes were placed into 1-ml wells of a 96-well plate. Cultures were incubated statically for 4 h at 37 °C, at which time 100 µL of SCFM2 or SCFM2 containing 20 µM HQNO (final concentration of 5 µM in cultures) was gently added to each culture. Cultures were either incubated statically or immediately mixed by vigorously pipetting for 1 min. After a 30-min incubation period, mixed cultures were again mixed by pipetting for 1 min. Tobramycin (256 µg/ml final concentration) or a sterile water control was then added to each well and incubated for 1 h before quantifying *S. aureus* by dilution plating onto MSA. Plates were incubated overnight before counting.

### CLSM Imaging.

Confocal microscopy was used to image mouse wounds infected with *dsRed*-expressing *S. aureus* and/or *gfp*-expressing *P. aeruginosa*. We photographed the objective over the wound each time a confocal image was taken, resulting in matched confocal images with wound region photographs. We then used the photograph to classify the image as core or edge, depending on the region of the wound being imaged. This allowed for exploration of the spatial structure at both the micron- and macro (mm)-scales. The local heterogeneity included vast areas without any visible bacteria as well as areas with highly dense bacterial populations. To capture this heterogeneity, the size of the confocal images varied depending on the area sampled and ranged from squares 250 μm by 250 μm (0.0625 cm^2^) to larger rectangles of up to 800 μm by 200 μm (0.16 cm^2^), which accounts for 1 to 3% of the total area of the wound. Each wound was imaged in three or more locations, and at least three wounds were imaged per condition, resulting in a total of 110 images.

Wounds were obtained 4 d post infection and cut in half, and one half was used for imaging while the other half was used for bacterial enumeration by CFU. Each wound was placed in a CoverWell™ Imaging Chamber Gasket, and two drops of ProLong Glass Antifade Mountant with NucBlue was added to prevent fading and minimize drift during imaging. Wounds were incubated for 30 min at 4 °C to allow the mounting medium to harden. Samples were then placed on the microscope with the surface of the wound facing the objective. All images were acquired using a Zeiss LSM 880 CLSM utilizing Zen image-capture software with 3 different detectors. Detection of *dsRed*-expressing *S. aureus* cells was performed with an excitation wavelength centered at 587 nm and an emission wavelength centered at 610 nm. Detection of *gfp*-expressing cells was performed using an excitation wavelength centered at 488 nm and an emission wavelength centered at 509 nm. Detection of DAPI was performed by exciting at 405 nm and detecting emission from 420 nm to 470 nm. All images were acquired using a 63× oil- immersion objective. The wound was scanned for fluorescence signals, and imaging began after centering in a region of interest. Images captured using tiles had 10% overlap and were later stitched using ZEN blue software. For biomass analyses, the biomass for each bacterial strain was calculated by adding the volume of voxels that were assigned to red or green fluorescence for *S. aureus* and *P. aeruginosa*, respectively. We included at least 2 fields of view per infection, and at least 3 infections per condition for all image analyses. Bacterial density was calculated for each image by dividing the biomass of bacteria in that image over the total volume of the image.

### Image Thresholding.

Confocal images were exported as a tiff stack and each channel was binarized using MATLAB (Simulink). Image analysis started using a histogram stretching routine to span the entire range of intensity values. These images were then passed through the Wiener filter routine, which identifies high contrast in a kernel and maintains it, while averaging low contrast areas. A threshold was then identified for each channel’s entire stack using Otsu’s method (62). Final images were generated by subtracting the red channel, corresponding to DsRed from the green channel, corresponding to GFP.

### PO.

PO was calculated as described previously (13, 15). In brief, the value from each image at each distance was calculated by binning the PO values, which range from 0 to 1, in intervals of 0.01 units and calculating the value for 50% of the population of 1,000 random samplings for PO. The mean value from each image per condition was reported as the final value.

### Enrichment Distance.

The highest value for PO from each sample across all distances was collected. In cases where more than one distance had the highest value, the weighted mean was calculated for the distance, using the population density of each distance as the weight. The reported enrichment distance was the mean per condition.

### RNA-sequencing.

Murine chronic wounds were generated as described above in 8 to 12 wk female C57/BL6 mice (Charles River) monoinfected with either *P. aeruginosa* PA14 or the isogenic *∆pqsL* mutant in duplicate. Wounds were excised and immediately added to RNA-later and prepared for RNA-sequencing as previously described (39, 63). rRNA was depleted using the QIAseq FastSelect kit (Qiagen) with bacterial and HMR mixed probes as per the manufacturer’s instructions. cDNA libraries were prepared using the NEBNext Multiplex small RNA library prep kit (New England Biolabs) as per the manufacturer’s instructions. Libraries were sequenced at the Molecular Evolution Core at the Georgia Institute of Technology by Illumina NextSeq500 75-bp single-end runs. Adapters were removed, and reads were trimmed using a minimum read threshold of 22 base pairs with Cutadapt version 2.6 (64). Reads were mapped to *P. aeruginosa* strain PA14 (accession number GCF_000014625.1) downloaded from the National Center for Biotechnology Information (NCBI) using Bowtie2 version 2.3.5 (65) and tallied with featureCounts version 2.0.1. Differential expression was determined with DESeq2 v1.36.0 (66) with betaPrior set to true.

## Supplementary Material

Appendix 01 (PDF)

## Data Availability

The raw sequencing files from this study are available at the NCBI Sequence Read Archive (SRA) under accession number PRJNA858071 and can be accessed at https://www.ncbi.nlm.nih.gov/bioproject/PRJNA858071 (67). Scripts for all image analysis and quantification are modified from Barraza et al. and are available at https://github.com/jupabago/PaSaChronicWounds. All study data are included in the article and/or *SI Appendix*.
