# Supplementary material for: Precise spatial structure impacts antimicrobial susceptibility of S. aureus in polymicrobial wound infections
Source: Proc Natl Acad Sci U S A. 2022 Dec 15;119(51):e2212340119. doi: 10.1073/pnas.2212340119 (PMC9907066; doi:10.1073/pnas.2212340119)
Supplement: Supplementary file 1 — Appendix 01 (PDF) [file pnas.2212340119.sapp.pdf]

PNAS

Supporting Information for

Precise spatial structure impacts antimicrobial susceptibility of *S. aureus* in polymicrobial wound infection

Carolyn B. Ibberson, Juan P. Barraza, Avery L. Holmes, Pengbo Cao, and Marvin Whiteley

Correspondence may be addressed to Carolyn B. Ibberson or Marvin Whiteley
Email: carolyn.ibberson@ou.edu
Email: mwhiteley3@gatech.edu

This PDF file includes:

Figures S1 to S5
Tables S1 and S2
SI References

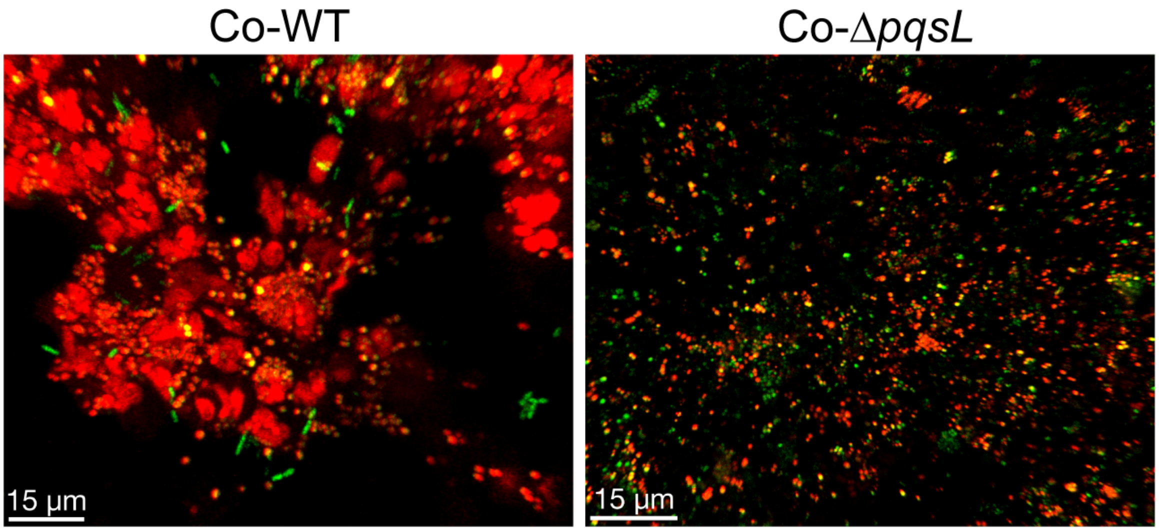

Fig. S1. Images of co-infected murine wounds. Confocal images of murine wounds co-infected with *S. aureus* and *P. aeruginosa* PA14 WT (Co-WT) or the isogenic Δ*pqsL* mutant (Co-Δ*pqsL*) at 4 days post-infection. *S. aureus* constitutively expresses DsRed and *P. aeruginosa* constitutively express GFP. Host cells (blue) were stained with NucBlue in the mounting medium.

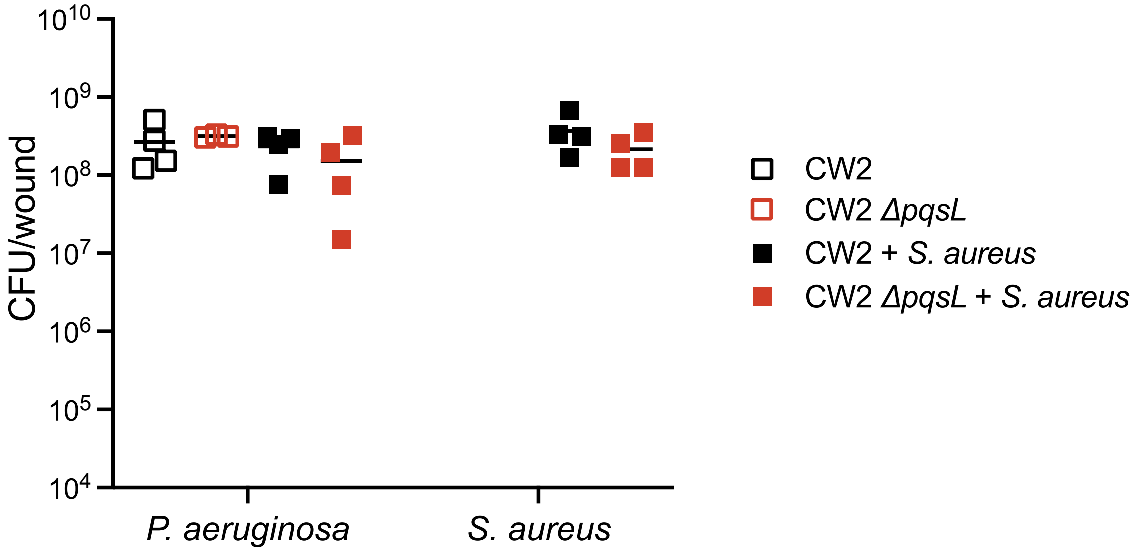

Fig. S2. *S. aureus* and a *P. aeruginosa* wound clinical isolate co-exist in a murine surgical wound model. Colony forming units of *P. aeruginosa* CW2-B1 or *S. aureus* in mono- (open squares) and co-infection (closed squares) with wildtype (black), or CW2-B1 *ΔpqsL* (red) in the murine wound model. Each symbol indicates a single animal infection.

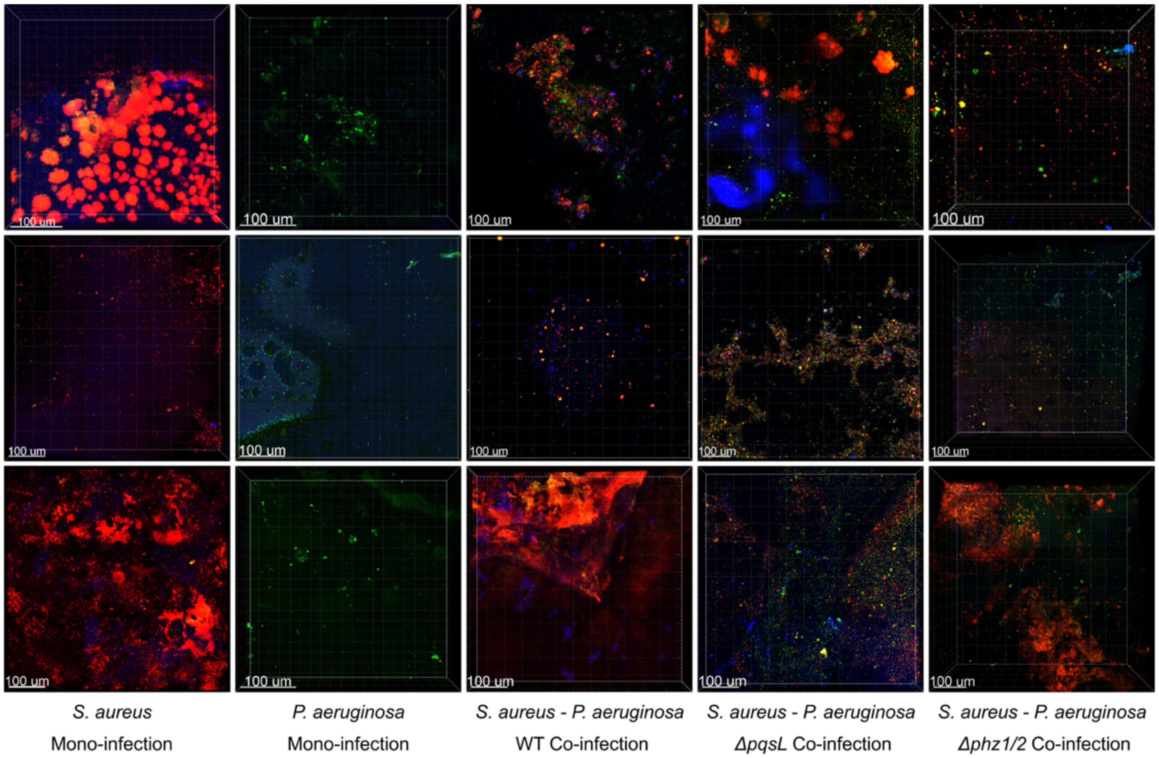

Fig S3. Patchy distribution of *S. aureus* and *P. aeruginosa* within wounds. *S. aureus* (red), *P. aeruginosa* (green), and host cells (blue) are shown. Images are representative of the range of observed spatial organization.

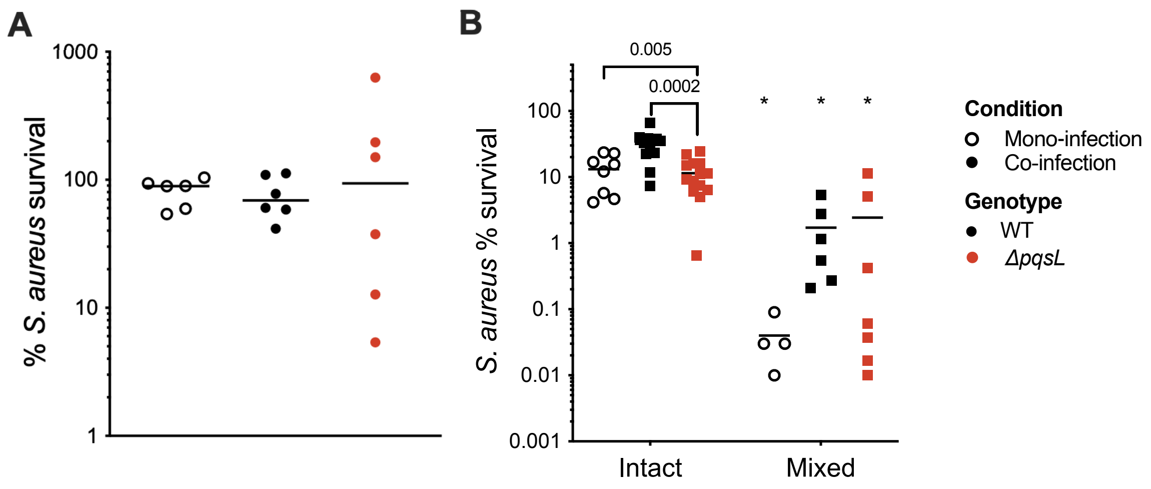

Fig. S4. *S. aureus* vancomycin and aminoglycoside tolerance in intact and mixed wounds. A) Percent survival of *S. aureus* to vancomycin in intact (structured) wounds compared to untreated portions of the same wound. Mono-infection is shown with open circles, co-infection with WT *P. aeruginosa* with closed circles, and co-infection with *P. aeruginosa ΔpqsL* with red circles. **B)** Percent survival of *S. aureus* to gentamicin in intact (structured) or mixed (homogenized) wounds compared to untreated portions of the same wound. Mono-infection is shown with open circles, co-infection with WT *P. aeruginosa* CW2-B1 with black squares, and co-infection with *P. aeruginosa* CW2-B1 *ΔpqsL* with red squares. All P-values were determined by a Mann-Whitney test, asterisks indicate $P < 0.01$ comparing Intact and Mixed wounds of each condition. Each symbol indicates a single animal infection.

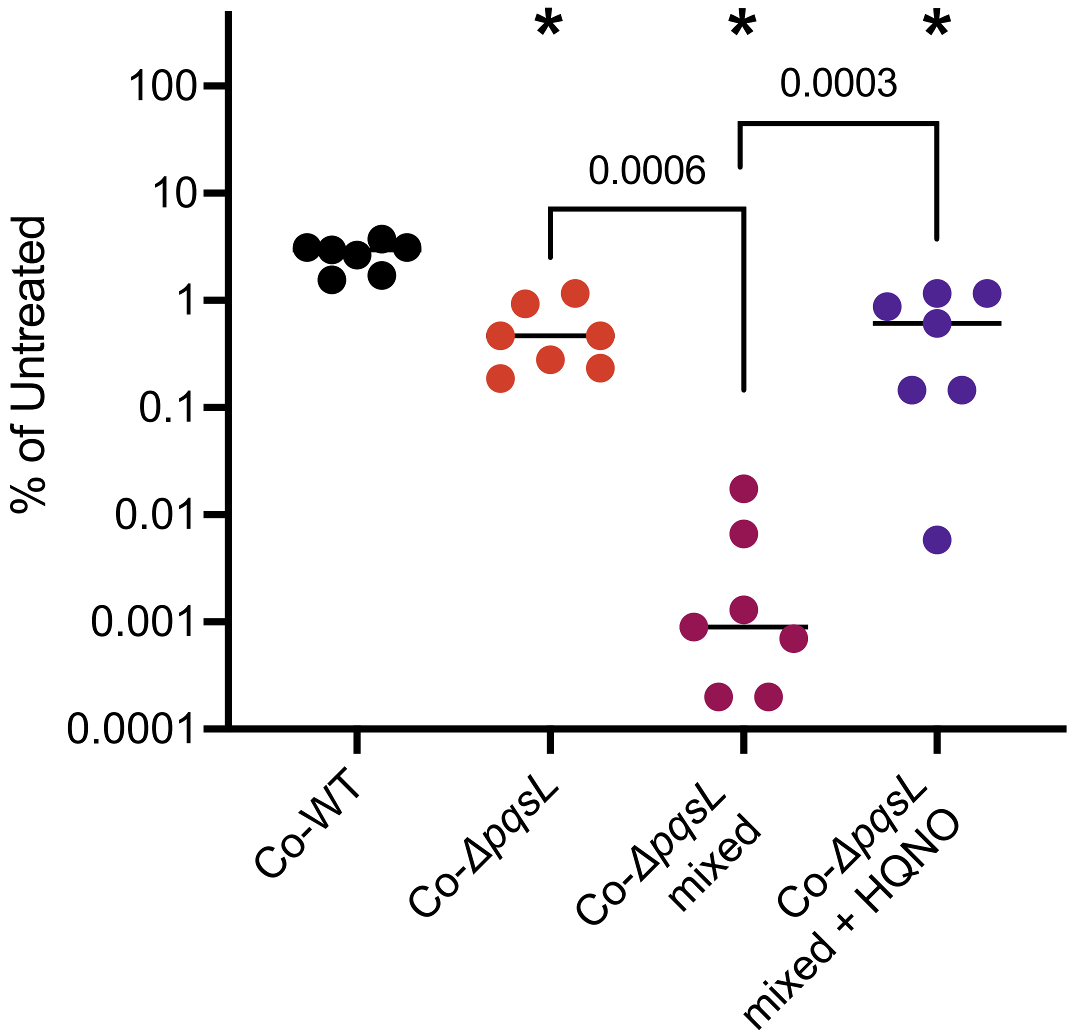

Fig. S5. *S. aureus* tolerance to the aminoglycoside tobramycin during co-culture in an in vitro CF infection model. Percent survival of *S. aureus* to tobramycin in vitro compared to untreated cultures. Co-infection with WT *P. aeruginosa* PA14 is shown with black circles (Co-WT), co-infection with *P. aeruginosa* PA14 *ΔpqsL* with red circles (Co-*ΔpqsL*), co-infection with *ΔpqsL* that has been vigorously mixed is shown in burgundy (Co-*ΔpqsL* mixed), and *ΔpqsL* with the addition of 5 µM HQNO prior to being vigorously mixed is shown in purple (Co-*ΔpqsL* mixed+HQNO). Statistical significance was determined with a Mann-Whitney test with a Benjamani, Krieger and Yeekutieli correction for multiple testing. Asterisks indicate an adjusted P-value ≦ 0.01 in comparison to Co-WT.

Table S1. Strains and plasmids used in this study.

Strain or plasmid	Description	Identifier	Source or Reference
RN4220 + pHC48	*S. aureus* RN4220 containing pHC48 which constitutively expresses dsRed	AH3865	(1)
LAC + pHC48	*S. aureus* LAC* (AH1263) + pHC48 which constitutively expresses dsRed	CI31	This study
LAC	*S. aureus* LAC* (AH1263) community-associated methicillin resistant USA300 isolate	CI3	(2)
PA14	*P. aeruginosa* strain PA14	CI132	(3)
PA14 *ΔpqsL*	PA14 with *pqsL* deleted	CI136	(4)
PA14 *Δphz1/2*	PA14 with *phz1* and *phz2* deleted	CI138	(5)
PA14 Tn7::GFP	PA14 with *gfp* at Tn7 site	CI143	This study
PA14 *ΔpqsL* Tn7::GFP	PA14 *ΔpqsL* with *gfp* at Tn7 site	CI157	This study
PA14 *Δphz1/2* Tn7::GFP	PA14 *Δphz1/2* with *gfp* at Tn7 site	CI154	This study
CW2-B1	*P. aeruginosa* strain CW2-B1	CI202	(6)
CW2-B1 *ΔpqsL*	CW2-B1 with pqsL deleted	CI203	This study
pBK-miniTn7-gfp2	*Escherichia coli* XL1-Blue + pBK-miniTn7-gfp2, constitutive GFP in miniTn7 transposon	AKN66	(7)
puxBF13	*E. coli* S17-1 + pUXBF13, transposase plasmid to allow for integration of miniTn7		(7)
pRK2013	*E. coli* + pRK2013, conjugative helper plasmid		(8)
pEXG2pq	Plasmid for allelic replacement of *pqsL*		(4)

Table S2. Differential expression by RNA-seq of operons adjacent to the *pqsL* locus in murine chronic wounds infected with *P. aeruginosa* wildtype or the *ΔpqsL mutant*.

Gene ID	Log2 Fold-Change	Adjusted *P*-value
bfiS	-0.83	0.88
bfiR	-0.19	1.00
PA14_09710	0.01	1.00
PA14_09730	NA	NA
PA14_09740	-2.64	0.16
PA14_09750	0.05	1.00
PA14_09760	-1.32	0.82

SI References

1. C. B. Ibberson, C. P. Parlet, J. Kwiecinski, H. A. Crosby, D. K. Meyerholz, A. R. Horswill, Hyaluronan modulation impacts *Staphylococcus aureus* biofilm infection. *Infect. Immun*. 84, 1917-1929 (2016).
2. B. R. Boles, M. Thoendel, A. J. Roth, A. R. Horswill, Identification of genes involved in polysaccharide-independent *Staphylococcus aureus* biofilm formation. *PLoS One* 5, e10146 (2010).
3. N. T. Liberati et al., An ordered, nonredundant library of Pseudomonas aeruginosa strain PA14 transposon insertion mutants. *Proc. Natl. Acad. Sci. U.S.A*. 103, 2833–2838 (2006).
4. J. P. Barraza, M. Whiteley, A *Pseudomonas aeruginosa* antimicrobial affects the biogeography but not fitness of *Staphylococcus aureus* during coculture. *mBio*. 12, e00047-21 (2021).
5. L. E. P. Dietrich, A. Price-Whelan, A. Petersen, M. Whiteley, D. K. Newman, The phenazine pyocyanin is a terminal signalling factor in the quorum sensing network of *Pseudomonas aeruginosa*. *Mol. Microbiol*. 61, 1308–1321 (2006).
6. A. da Silva, "*Understanding the diversity and evolution of complex wound infections*" (University of Nottingham, Nottingham, UK, 2019).
7. B. Koch, L.E. Jensen, O. Nybroe, A panel of Tn7-based vectors for insertion of the *gfp* marker gene or for delivery of cloned DNA into Gram-negative bacteria at a neutral chromosomal site. *J Microbiol Methods.* 45, 187-195 (2001).
8. D. H. Figurski, D. R. Helinski, Replication of an origin-containing derivative of plasmid RK2 dependent on a plasmid function provided in trans. *Proc. Natl. Acad. Sci. U.S.A*. 76, 1648–1652 (1979).
